# Experiences of Person‐Centred Care Among Nurses in COVID‐19 Wards: A Qualitative Study

**DOI:** 10.1002/nop2.70293

**Published:** 2025-08-21

**Authors:** Myoungsuk Kim, Yongmi Lee, Hyun‐Ju Kang

**Affiliations:** ^1^ College of Nursing Kangwon National University Chuncheon‐Si Republic of Korea

**Keywords:** COVID‐19, pandemic, person‐centred care, qualitative research

## Abstract

**Aim:**

While there is extensive research on nurses' experiences in caring for COVID‐19 patients, studies focusing on person‐centred care in this context remain limited. This study aimed to obtain a thorough understanding of the meaning and essence of person‐centred care experienced by nurses in COVID‐19 wards in South Korea.

**Design:**

This study adopted a qualitative research design.

**Methods:**

Data were collected from April 2022 to February 2023 using one‐on‐one in‐depth interviews with nine nurses working in COVID‐19‐designated hospitals; these data were analysed using content analysis. This study adhered to the Consolidated Criteria for Reporting Qualitative Research (COREQ) guidelines.

**Results:**

The five categories were: *encountering barriers between nurses and patients, the conflict between principles and dignity, providing individualised care even under controlled circumstances, providing care in a therapeutic community relationship and desiring stable care even in a crisis*.

**Conclusion:**

This study examined nurses' challenges and experiences in delivering person‐centred care during the COVID‐19 pandemic. Despite the conflict between infection control guidelines and individual patient needs, nurses sought to address patients' psychological and physical challenges while overcoming COVID‐19 together, leading to satisfaction for both patients and nurses. Systemic support and education are essential to sustain person‐centred care, and this study provides valuable insights for improving nursing practice in crisis situations.

**Patient or Public Contribution:**

No patient or public contribution.

## 1 Introduction

The World Health Organization (2022) announced the outbreak of the COVID‐19 pandemic in March 2020, while approximately 760 million cases and 6.8 million deaths were confirmed by 2022. The global pandemic posed several challenges and sudden changes for healthcare systems worldwide (Safdari et al. 2023). The South Korean healthcare system responded immediately to the COVID‐19 pandemic with measures such as establishing strict quarantine and social‐distancing protocols, setting up screening clinics, and managing COVID‐19 hospitals based on regional needs (Chung et al. 2022) for the treatment of high‐risk patients (Shin and Cheon 2021). Patients hospitalised owing to COVID‐19 experienced deterioration of their physical and psychological well‐being, frequently facing stress and depression. Therefore, in South Korean hospitals, these patients were isolated and treated individually, which led to a sudden disruption of their social relationships (Lee et al. 2021). Nurses caring for patients in COVID‐19 wards faced a unique, novel and challenging situation. Thus, medical staff in COVID‐19 wards responded by not only providing support for the treatment and examination of COVID‐19 patients but also paying increased attention to self‐quarantine practices (Oh and Lee 2021).

Nurses play a crucial role in healthcare systems' response to pandemics. During such crises, when discharging their duties, nurses experience concerns and fears regarding their safety and that of their families (Fernandez et al. 2020). This was the case for nurses during the COVID‐19 pandemic; accordingly, there has been growing attention to nurses' experiences and challenges on the front lines of the pandemic. Nurses in COVID‐19 wards commonly reported work overload and psychological stress, including fear of infection and anxiety (Kackin et al. 2021). Furthermore, they faced difficulties stemming from organisational weaknesses, including insufficient medical knowledge and understaffing (Karimi et al. 2020). Despite these challenges, nurses reported that caring for COVID‐19 patients, making personal sacrifices, and maintaining a strong sense of professional responsibility were rewarding (Chung et al. 2022; Robinson and Stinson 2021). Several studies have examined nurses' experiences in providing care for COVID‐19 patients during the pandemic. Firouzkouhi et al. (2022) conducted a systematic review of studies on nurses' experiences during the COVID‐19 pandemic, identifying key themes such as nursing's weaknesses and strengths at the beginning of the pandemic, nursing beyond challenges related to the pandemic and family and career challenges. Previous studies have explored various aspects of nurses' experiences during the COVID‐19 pandemic; however, there is a pressing need to gain a deeper understanding of how nurses coped with the aforementioned challenges while adhering to the principles of person‐centred care. Moreover, studies examining nurses' experiences in providing person‐centred care in South Korea's healthcare system are particularly scarce.

The concept of person‐centred care emerged from the understanding that nursing care should go beyond a single‐minded focus on diseases and medical personnel, considering patients' opinions and preferences (Edvardsson 2015). Recent studies on person‐centred care have highlighted its main attributes: considering patients' individuality and dignity, engaging in decision‐making through effective communication and fostering a comfortable environment at the organisational level (Lee et al. 2020). Recently, it has been suggested that person‐centred care should be balanced to support patients' social participation, mental health and quality of life under quarantine and social‐isolation conditions during the COVID‐19 pandemic (Dichter et al. 2020).

Challenges such as staff shortages and exhaustion pose barriers to person‐centred care (Morgan et al. 2023), which aims to provide individualised care by establishing a therapeutic relationship with the patient, respecting and actively responding to their unique needs, and ensuring their authority and autonomy in their treatment decisions (Lusk and Fater 2013).

Some studies have examined person‐centred care during the COVID‐19 pandemic. A positive correlation was found among South Korean hospitals' infection‐control culture, nurses' empathy levels in COVID‐19 wards, and the degree of person‐centred care (Yun and Jeon 2022). Moreover, the more developed nurses' communication ability, clinical experiences and awareness of the nursing profession and working environments, the higher their degree of person‐centred care (Jung and Kim 2022). Although prior studies have investigated the factors affecting the person‐centred care of nurses in COVID‐19 wards, research on their experiences and how they provide person‐centred care is alarmingly scarce. In Sweden, intensive‐care‐unit nurses exhibited high person‐centred care intention. However, because of the overwhelming focus on COVID‐19, providing person‐centred care was found to be challenging in practice (Andersson et al. 2022). In China, medical staff were reported to effectively communicate with older‐adult COVID‐19 patients to better understand their needs and offer comfort and support (Li et al. 2021). Despite the COVID‐19 pandemic, healthcare providers for patients with dementia were able to provide person‐centred care by restructuring care services and changing or adjusting their perceptions of person‐centred care (Martin 2022). However, to the best of our knowledge, no studies have examined person‐centred care from the perspective of nurses who cared for COVID‐19 patients exclusively. Particularly, it is essential to understand how nurses perceived and experienced the changes brought about by the COVID‐19 pandemic in delivering person‐centred care, including emotional challenges, communication barriers and organisational support's impact.

Without a deep understanding of nurses' person‐centred care, it will be difficult to ensure that person‐centred care is provided in a balanced manner in clinical practice if other pandemics occur. Establishing this understanding will serve as a foundation for delivering person‐centred care during such crises. Therefore, this study aims to provide an in‐depth understanding of nurses' experiences in providing person‐centred care in COVID‐19 wards in South Korea, highlighting the complexities and challenges they faced while striving to maintain a person‐centred approach amidst unprecedented circumstances.

## 2 Methods

### 2.1 Study Design

This qualitative study used content analysis to understand the person‐centred care experiences of clinical nurses in COVID‐19 wards in South Korean hospitals. Content analysis is a qualitative method commonly used in healthcare research to understand specific phenomena. It efficiently and systematically classifies qualitative interview data, thereby enabling an understanding of both the content and contextual meaning (Hsieh and Sarah 2005).

### 2.2 Sampling and Recruitment

The research population consisted of nurses working in COVID‐19 wards at hospitals in Chuncheon and Gangneung City, Gangwon Province, in South Korea. Purposive sampling was used to select participants based on their years of work experience and the length of their experience in COVID‐19 wards, ensuring they could provide detailed information, thus aligning with this study's objectives. Inclusion criteria required participants to be registered nurses with at least 6 months of experience caring for patients in COVID‐19 wards. To recruit participants, initial cooperation was obtained from the hospital nursing department, while participants were introduced by a nurse working in a COVID‐19 ward. Nurses who understood the study's purpose and voluntarily consented to participate were included.

Nine nurses (seven women and two men) participated. The Chuncheon hospital's COVID‐19 ward comprised 22 nurses (17 women and five men), while that of the hospital in Gangneung had a total of 48 nurses (42 women and six men). Among the participants, five were staff nurses and four were charge nurses. The participants' ages ranged from 27 to 41 years (mean = 32.1), while total nursing experience ranged from 2 years and 6 months to 17 years. Participants' average nursing experience in COVID‐19 wards was 17.8 months (Table 1).

**TABLE 1:** Participants' general characteristics.

No.	Sex	Age (year)	Education level	Religion	Marital status	Position	Total clinical experience	Experience of caring for COVID‐19 patients	Original working ward
1	Female	27	Bachelor	None	Single	Staff nurse	Two years, six months	Eight months	Haematology‐Oncology ward
2	Female	41	MSN	None	Married	Charge nurse	17 years	Two years	ICU
3	Female	32	MSN	Buddhism	Single	Charge nurse	11 years	One year, six months	Pulmonary ward
4	Male	29	MSN	None	Single	Charge nurse	Four years, one month	Seven months	Gastrointestinal ward
5	Female	35	MSN	None	Single	Staff nurse	13 years	One year	ICU
6	Male	32	Bachelor	Catholic	Single	Staff nurse	Four years, 10 months	One year	ICU
7	Female	38	MSN	Protestant	Married	Charge nurse	17 years, two months	Four years, two months	Paediatric ward
8	Female	28	Bachelor	None	Single	Staff nurse	Five years, five months	Two years, four months	ICU
9	Female	27	MSN	None	Single	Staff nurse	Five years	Two years	Pulmonary ward

Abbreviations: ICU, intensive care unit; MSN, Master of Science in Nursing.

### 2.3 Data Collection

From April 2022 to February 2023, three researchers (female nursing professors with doctoral degrees in nursing) conducted one‐on‐one in‐depth interviews with the participants to collect data. Individual interviews took approximately 60–90 min. Due to the COVID‐19 pandemic, interviews were conducted online via Zoom software to minimise the risk of infection. These online interviews were set up to simulate face‐to‐face interaction by maintaining engagement through clear communication and visual contact. These online interviews were conducted in a quiet place to create a comfortable atmosphere for participants to share their experiences. The participants were in quiet locations, such as break rooms or their homes, with restricted access to others. Before starting the online interviews, participants were asked to provide their consent, and written consent forms were submitted via email.

At the start of each interview, participants were given a brief explanation of the research topic. Interviews began with general questions about daily life and hospital experiences, gradually progressing to research‐specific questions. The interview questions were developed through literature reviews and discussions among the researchers to ensure they aligned with the study's objectives. A pilot interview was conducted with a nurse who had experience caring for COVID‐19 patients to determine if any modifications were needed for the interview questions; however, no adjustments were necessary. The main question and sub‐questions were the following:

Main question:

- “Tell me about your experiences with person‐centred care while caring for COVID‐19 patients.”

Sub‐questions:

- “How does nursing care for COVID‐19 patients differ from your previous nursing experiences?”
- “What significance did the experience of person‐centred care in COVID‐19 patient care have for you?”
- “Could you share your thoughts on person‐centred care?”

Follow‐up questions were asked as needed to clarify ambiguities or seek further explanation from the participants, such as, “Could you elaborate on that situation?” Or “Could you provide an example?” Data collection continued until saturation was achieved, concluding after interviews with nine nurses when the data became repetitive and no new statements, concepts, or topics emerged.

### 2.4 Data Analysis

Data were analysed using content analysis. All steps were performed manually, with each step carried out systematically as follows:

- First, researchers familiarised themselves with the data by reading the statements several times.
- Second, significant words, sentences and paragraphs were identified, and their meanings were interpreted and explained.
- Third, similar meanings were grouped and coded, and the meanings derived from each code were repeatedly checked.
- Finally, the derived meanings were collected to identify subcategories, while main categories were derived by grouping similar, non‐overlapping codes.

To enhance the validity and reliability of the data analysis, data were independently analysed by three researchers, and discussions continued until an agreement regarding the subcategories and categories was reached. The researchers read the transcripts several times to understand participants' meaning. Each researcher individually analysed the data; subsequently, the researchers reviewed and discussed their opinions.

### 2.5 Rigour

To increase reliability, interviews were recorded to prevent any missing data. After the analysis, transcriptions and analysis results were sent to each of the participants by e‐mail to confirm whether they matched their statements. Data were collected until the participants' statements emerged repeatedly due to data saturation.

To increase dependability and reduce differences, the researchers reviewed the analysed data until they reached a consensus. Confirmability was strengthened by researchers' continuous reflection throughout the research process to exclude biases, while all researchers checked whether their subjective thoughts influenced data interpretation. Additionally, the Consolidated Criteria for Reporting Qualitative Research (COREQ) reporting guidelines were adopted (Tong et al. 2007) to ensure unbiased reporting.

### 2.6 Ethical Considerations

Approval for the study was obtained from the Institutional Review Board of [Kangwon National University]. Prior to data collection, the researchers obtained participants' written consent. The research purpose, procedures and potential uses were explained to the participants, and they were informed of their right to decline participation or withdraw from the study at any point. Participants also consented to audio/video‐recording of interview proceedings. The collected data were immediately coded and stored to ensure anonymity. After the interview, the participants received a gift certificate as an expression of gratitude.

## 3 Results

As a result of analysing the person‐centred care experience data of nurses in the COVID‐19 ward, open codes (*n* = 101) and formulated meanings were merged into 12 subcategories, while five categories were formed by combining these subcategories (Table 2).

**TABLE 2:** Categories and subcategories of participants' experiences.

Categories	Subcategories	Formulated meanings
Encountering barriers between nurses and patients	Difficulty getting close to patients due to a vague anxiety	· Reluctance to contact patients due to fear of infection · Experiencing difficulties in patient care due to insufficient knowledge about COVID‐19 infection · Perception of threats to personal life and safety during patient care
	The physical environment barriers to care	· Difficulty in communicating with patients due to noise and the use of protective equipment · Physical discomfort from protective equipment interferes with nursing care · Difficulty in approaching patients easily due to spatial separation caused by negative‐pressure systems
The conflict between principles and dignity	No choice but to accept the principle of saving lives first	· Uncertainty of COVID‐19 limits patients' autonomy · Nursing care is provided strictly according to the COVID‐19 patient‐management protocols and manuals · Prioritising life‐saving treatments over other considerations in critical situations · Perceiving person‐centred care as challenging in life‐threatening patient situations
	Feeling the limits of nursing within restricted guidelines	· Limited to providing only basic care according to restricted protocols · Experiencing conflict between adhering to protocols and meeting individual patient needs · Questioning the uniform nursing care that disregards individual patient needs
	Feeling powerless owing to a failure to help patients during their final moments	· Feeling discouraged by the reality of maintaining only prescribed duties until the end of a patient's life · Unable to provide proper end‐of‐life care due to adherence to manuals
Providing individualised care even under controlled circumstances	Trying to fulfil limited basic needs	· Striving to meet patient needs within the limitations of isolation environments · Assisting patients with daily activities they are unable to perform independently in isolation settings · Providing routine nursing care to meet patients' basic needs during isolation
	Understanding patients through empathy	· Striving to understand patients' discomfort and fears from their perspective · Attempting to understand patients' feelings of resentment and frustration · Identifying individual needs by attentively listening to patients' words and observing their actions
	Acting as a bridge between patients and families	· Acknowledging family members' feelings and attending to their needs · Maintaining regular contact with families to share the patient's condition and provide emotional support · Striving to alleviate patients' anxiety through continuous interaction with family members
Providing care in a therapeutic community relationship	Shared feelings being present through emotional support	· Striving to provide support by sharing the patient's emotions and being present · Understanding the patient's anxiety and loneliness and being present with them · Encouraging patients to express their emotions
	Satisfaction obtained from interacting with patients	· Gaining strength through collaborative efforts with patients to overcome COVID‐19 · Providing communication and care founded on rapport · Efforts to engage in active communication for mutual understanding and emotional exchange
Desiring stable care even in a crisis	Desire for a person‐centred environment	· Recognising the need to establish a person‐centred psychosocial support system · Demand for developing standardised work guidelines to implement person‐centred care · Hoping to create an environment for the provision of quality nursing care through securing adequate nursing staff · Anticipating the development of tailored protocols and establishment of educational systems centred on patient needs · Recognising the need for the development of a systematic educational program to enhance competencies in person‐centred nursing care
	Thoughtful mindset to understand patients	· Recognising the necessity of fostering a mindset oriented toward person‐centred nursing · Providing care with respect, treating the patient as a person rather than a confirmed case · Seeking to establish a mutually respectful therapeutic relationship grounded in patient dignity

### 3.1 Encountering Barriers Between Nurses and Patients

#### 3.1.1 Difficulty Getting Close to Patients due to a Vague Anxiety

The participants felt vague anxiety about COVID‐19 due to a lack of knowledge of the disease. Consequently, nurses were hesitant to provide even basic care. They stated that they could not think of the need for person‐centred care because their own safety was uncertain.

As COVID‐19 was a novel infectious disease, my safety was crucial, and there may have been heightened fear. While providing nursing care, I had numerous vague thoughts about whether I would be infected, so I thought about my safety first, not that of the patients, when I was caring for them.

(Participant 7)

As a nurse, I was also afraid. I was scared. I did not know much about this disease, so I did not want to get infected. Thus, I tried to avoid close contact with my patients as much as possible, although I should not have. I am sure the patients must have felt that I was avoiding them. I am sure they knew.

(Participant 8)

#### 3.1.2 The Physical Environment Barriers to Care

Participants stated that the different nursing environment was also a major constraint in providing person‐centred care. It was challenging to move around because they had to wear mandatory protective equipment, such as powered air purifying respirators (PAPRs), and even basic conversations were impossible due to the mask and the noise of machines in the room.

I cannot hear well. When I wear the PAPR and turn it on, I hear a constant buzzing sound. Since we installed a negative‐pressure facility, there is a constant loud noise in the room from the fan running. Therefore, there may be adverse effects due to the noise.

(Participant 4).

I just felt like all communication was completely blocked. Due to the PAPR and N95 masks. When people talked, they relied not only on verbal communication but also non‐verbal cues. If they were unable to hear well, they tried to infer the message by looking at the shape of the mouth. Since I was wearing a mask when I was talking to the patients, sometimes we could not hear or understand each other.

(Participant 8)

### 3.2 The Conflict Between Principles and Dignity

#### 3.2.1 No Choice but to Accept the Principle of Saving Lives First

Participants stated that in nursing care in the past, patient care not only focused on improving the patient's condition but also on their individual needs. However, because COVID‐19 is a severe and potentially life‐threatening disease, they prioritised saving the patient's life and improving their symptoms. They also mentioned that the uncertainty of the disease resulted in limitations to the patient's autonomy and choice.

Strangely, COVID‐19 patients seem to recover very slowly, and there are not many cases in which they have improved once their condition worsened. Thus, I could not help but think about how to help the patients get better. The most important thing is to prioritize treatment because, to do anything else, one must first be alive. Therefore, the therapeutic approach takes precedence.

(Participant 4)

When a patient becomes irritable, sometimes the only option we have is to physically restrain them using restraints. I understand their situation, but there is a greater risk if they were to fall.

(Participant 6)

#### 3.2.2 Feeling the Limits of Nursing Within Restricted Guidelines

Participants expressed that intensive care‐oriented treatment was conducted during the COVID‐19 pandemic; they identified the limitations of nursing in a treatment‐oriented environment that delivers nursing according to guidelines rather than based on individual patients' needs. They also wanted to meet the needs of each patient and provide person‐centred care but questioned the uniform treatment provided to all patients and felt conflicted as to whether it was appropriate to prioritise saving lives over providing person‐centred care.

The family members grabbed my hand and cried, asking, “We could not even hold a funeral. Oh! What should we do?” Should I not cry? … Did I really have to respond strictly according to the manual? At that moment, I felt so powerless and so weak, having such thoughts.

(Participant 2)

There were senior nurses who, when their junior colleagues provided person‐centred care, would ask, “Can you handle that? Can you take responsibility for your actions? Are you capable of taking responsibility for your actions? If a patient were to go to the restroom or if a patient were to fall, can you take responsibility?”

(Participant 5)

#### 3.2.3 Feeling Powerless Owing to a Failure to Help Patients During Their Final Moments

Participants expressed they could not provide proper person‐centred care because of the need to avoid the transmission of the infection, even as their patients' condition worsened or they died. In addition, they stated feeling powerless because of the mandate to follow strict procedures, which could not even protect the dignity of the patient in their final moments.

When providing end‐of‐life care, I felt terrible. In general wards, during the process of patients' death, the doctor would come and explain the prognosis, while family members would be present to comfort the patient and express their final wishes… Time would be allowed for them to accept their death. However, in COVID‐19 wards, family members could only visit after the patient passed away, and even virtual visits through CCTV were limited to around 10 minutes. Additionally, the waiting period for them to take a COVID‐19 test resulted in a loss of time.

(Participant 1)

The most difficult thing for me was witnessing the end‐of‐life stage of a COVID‐19 patient. It was truly heart‐breaking to know that during the patient's final moments, their loved ones could only watch through CCTV. How would the families feel as they watched their loved ones' end‐of‐life through CCTV?

(Participant 4)

### 3.3 Providing Individualised Care Even Under Controlled Circumstances

#### 3.3.1 Trying to Fulfil Limited Basic Needs

Participants felt sorry for patients because, from the moment they were diagnosed with COVID‐19 and quarantined, they had to surrender their autonomy. Thus, they made efforts to address patients' basic needs and prioritise their demands within the limits of what was permitted.

Some patients found it very difficult because they could not brush their teeth. Thus, we made sure to provide them with disposable toothbrushes and toothpaste. However, we also had to bring them water to rinse their mouths after brushing their teeth. Due to the need for patients to rinse their mouths with water and spit it out, we had to provide them with extra disposable cups. Although the task may have seemed trivial, it required considerable effort; it made the patients feel better.

(Participant 2)

During my conversations with patients' family members, I often said, “Please bring the patient his/her favorite food or snacks.” Although it may seem like a small thing because the patients were quarantined, they felt great joy! I paid close attention to these details.

(Participant 3)

#### 3.3.2 Understanding Patients Through Empathy

Participants mentioned that while caring for patients, they often wondered, “What if I were in the patient's shoes?” They looked at things from the patients' perspective and realised what had to be solved by empathising with the patient. They mentioned being present with the patient and understanding the emotions they felt most intensely, such as fear.

I am feeling extremely anxious right now. This is my first time experiencing a situation like this; thus, coming to the hospital makes me very anxious. Some patients are crying constantly. They may feel anxiety about the new environment, about being in a confined space, and about death. Feelings of resentment may have emerged, and there may hold resentment toward family members. Perhaps there could be feelings of self‐blame, wondering “Why did I get infected?”

(Participant 4)

I wanted to make the patients feel a little less lonely. No matter what the situation was, as an observer, I understand it better than anyone else. As I also have grandparents, I could empathize well with such situations. I wanted to be a nurse who could help patients develop trust, feel supported, and not feel lonely or scared even if they were alone. I wanted to provide nursing care that patients could rely on both therapeutically and emotionally.

(Participant 9)

#### 3.3.3 Acting as a Bridge Between Patients and Families

Participants understood that patients longed to see their loved ones and felt frustrated about being separated from their families. Participants stated that they also tried to solve this problem because patients' families also felt anxious because they did not know the condition of the patients. The participants also mentioned that they tried to understand the psychological state and difficulties of not only the patients but also their families and made efforts to support and assist the families as well.

After receiving calls from the family members, when entering the negative‐pressure room, we would convey messages such as, “Your family contacted us today and wanted to let you know that they love you. They want you to keep doing well and stay strong.” I thought they would not remember. However, later, they mentioned that they did remember. “Last time when someone came and told me what my family had said, it gave me some strength.”

(Participant 6)

When family members called and asked if the patients felt hot or cold due to the negative‐pressure room and if there was anything else they needed, I paid attention to these things, even if it was something simple.

(Participant 9)

### 3.4 Providing Care in a Therapeutic Community Relationship

#### 3.4.1 Shared Feelings Being Present Through Emotional Support

The participants stated that for person‐centred care, respectful interaction with the patient and helping the patient recognise their presence as a means of support are crucial actions.

Because I had a strong belief that we needed to overcome COVID‐19 together, I expressed my emotions by saying to patients “We must complete this treatment together,” even if we could not fully explain everything.

(Participant 3)

#### 3.4.2 Satisfaction Obtained From Interacting With Patients

While providing person‐centred care, participants felt satisfaction and pride in their work through patients' positive reactions.

When a strong rapport was established with the patient and they were discharged from the hospital, they often said, “I have really received great treatment” and “I am grateful,” while holding my hands or giving me a hug. I felt very proud of myself. They understood and appreciated my sincerity.

(Participant 3)

A patient underwent a tracheostomy and after waking up, wrote a letter asking if I had not been burdened because of him. He wrote a letter to me! That is why I have a vivid memory of that person.

(Participant 6)

### 3.5 Desiring Stable Care Even During Crises

#### 3.5.1 Desire for a Person‐Centred Environment

The participants believed that to provide proper person‐centred care, tangible support is crucial. In other words, they not only felt the need to ensure proper staffing but also to establish a system for person‐centred care, creating manuals and providing practical education, as they experienced difficulties in providing nursing care on an ad‐hoc basis, without a proper system. They also stated the need for practical improvements in the working environment.

Because it is not structurally established, everything is being done temporarily. I think it would be beneficial to have some improvements in terms of the system, facilities and environment; it would be better if nurses and healthcare providers received education or participated in workshops to address these aspects.

(Participant 5)

If we have manuals and systems that guide us on how to provide care in certain ways, it would help us approach person‐centred care more effectively.

(Participant 8)

#### 3.5.2 Adopting a Thoughtful Mindset to Understand Patients

The participants emphasised that to provide person‐centred care during a crisis, it is necessary to not only respect each patient as an individual but also communicate effectively with patients, even if a system and staffing are adequate. Nurses who possess these qualities could provide person‐centred care tailored to the patient's situation and environment.

Even if the staffing issue is resolved, if nurses have the mindset to provide one‐on‐one care, this can have a significant impact. Even with education, there is a difference between individuals who possess such a mindset and those who do not.

(Participant 3)

## 4 Discussion

This study explored nurses' experiences of person‐centred care in COVID‐19 wards using content analysis, through which five categories were identified: *encountering barriers between nurses and patients; the conflict between principles and dignity; providing individualised care even under controlled circumstances; providing care in a therapeutic community relationship; and desiring stable care even in a crisis*.

In the category *encountering barriers between nurses and patients*, participants mentioned not even considering person‐centred care during the early stages of the pandemic due to fears and anxieties about infection and environmental difficulties, such as wearing protective equipment and being exposed to the noise in negative‐pressure isolation rooms, as indicated in previous studies (Chung et al. 2022; Fernandez et al. 2020; Kim 2021; Kim et al. 2022; Noh et al. 2021; Oh et al. 2021). A previous systematic literature review revealed that nurses experienced knowledge deficiencies and insufficient preparedness in managing infectious diseases during the COVID‐19 pandemic (Firouzkouhi et al. 2022), which increased their anxiety and fear in caring for patients. A qualitative study on nurses' experiences during the COVID‐19 pandemic found that nurses adopted inappropriate strategies such as avoiding contact, excessively adopting protective measures, or displaying aggression toward patients and their companions who ignored protective measures (Manookian et al. 2024; Shahmari et al. 2024), which highlights the heightened anxiety that nurses experienced owing to the fear of infection. Such anxiety can become a barrier to providing person‐centred care (Morgan et al. 2023), emphasising the need to develop coping strategies to reduce nurses' anxiety. Conversely, while the use of protective equipment and negative‐pressure isolation rooms is important to prevent the transmission of infection, these factors also made effective communication with patients more difficult. Previous research (Andersson et al. 2022) revealed that masks act as a barrier to communication, leading to the avoidance of talking points other than COVID‐19‐related issues. However, despite the various challenges posed by the pandemic, person‐centred communication plays a significant role in building trust with patients, improving the quality of nursing and promoting patients' psychological well‐being (Li et al. 2021). Despite the environmental difficulties during the pandemic, this theme highlights the need to enhance strategies that promote effective communication, a key element of person‐centred care.

The category *the conflict between principles and dignity* was not observed in previous domestic studies on the experiences of nurses caring for COVID‐19 patients (Kim 2021; Lee and Song 2021; Noh et al. 2021; Oh et al. 2021). However, qualitative research conducted in another country (Andersson et al. 2022) revealed that nurses reported inappropriate person‐centred approaches and described nursing as repetitive and dehumanising, likening it to working in a factory. Similarly, another qualitative study (Manookian et al. 2024) revealed findings related to nurses' ethical conflicts, as participants reported changing their approach to provide patient care prioritising caution to prevent infection transmission while experiencing a conflict between self‐care and comprehensive patient care, and inadequate care. In our study, participants experienced frustration and powerlessness as they struggled to provide treatment‐centred care, were unable to meet patients' individual needs, and were unable to uphold patients' dignity during end‐of‐life care due to the need to adhere to infection‐control guidelines. Moreover, they experienced internal conflicts when they were unable to provide appropriate support to families who could not be with the patients during their final moments. This is similar to prior qualitative research findings, in which participants expressed regret because nothing could be done during the patient's death from COVID‐19 (Williams et al. 2024). Other studies highlighted challenges in maintaining human dignity during the COVID‐19 pandemic, including patients having to face death alone and relatives or family members being left with no time to prepare for their farewells (Becqué et al. 2022). Ensuring that patients facing death can do so with dignity is the most important goal in end‐of‐life care. Even during a pandemic, virtual farewells between patients and their families should be ensured and social workers and clergy should assist in the grieving process (Rao and Kelemen 2021). Systemic changes and strategies are required for end‐of‐life care to not only allow patients to experience a dignified death but also address the conflicts experienced by nurses. In a similar context, Manookian et al. (2024) identified the sub‐theme of *prioritising life* under the theme of *ethical conflicts* in a qualitative study. Their study reported that participants faced situations where they had to prioritise patients based on age or medical condition due to resource shortages. In this process, older patients were often overlooked, while younger patients received priority, leading to psychological stress and ethical conflicts between professional obligations and practical realities. Conversely, our study did not observe such conflicts but instead identified a tension between the obligation to adhere to infection‐prevention principles and the inability to meet patients' needs or uphold their dignity, thus highlighting a difference from previous research.

In the category of *providing individualised care even under controlled circumstances*, despite limitations such as excessive workload, staff shortages, and the need to wear protective equipment, participants gradually developed an understanding of patients' difficulties and were eager to address individual needs. They acknowledged the psychological challenges patients experienced due to isolation, such as frustration, loneliness, anxiety, and fear, as well as the physical discomfort related to pain and hygiene. Previous research (Li et al. 2021) has shown that COVID‐19 patients experience negative emotions and depression due to fear of death, social isolation, and separation from their families. In the present study, participants made efforts to frequently engage in conversation, actively listen, address patients' needs, and serve as a link between patients and their families, despite their heavy workloads and challenging auditory conditions caused by protective equipment. This result is similar to the findings of a previous qualitative study (Li et al. 2021), which showed that healthcare professionals facilitated contact between patients and their families through phone calls, video chats and the delivery of encouraging messages, thereby ensuring a connection. However, this differs from the findings of a prior study (Andersson et al. 2022), which reported difficulties in person‐centred care during the COVID‐19 pandemic, such as communication challenges due to protective masks and a lack of holistic approaches to prevent the spread of the virus. Conversely, our study found that even in such situations, nurses made efforts to understand patients' difficulties and, despite the challenges, tried to provide individualised care by identifying their specific needs.

The category *providing care in a therapeutic community relationship* includes nurses' growth and development through mutual interaction with patients. Initially, nurses provided care to patients in a unidirectional manner; however, a sense of collectively overcoming COVID‐19 emerged gradually. Nurses were able to find fulfilment and satisfaction in their work. Despite their excessive workloads and busy schedules, they increased the time spent with patients and made efforts to build relationships through interaction and emotional support. This finding is similar to that of a previous study (Li et al. 2021), which found that healthcare professionals interacted with patients, formed relationships, and provided emotional support despite heavy workloads. However, our findings differ from those of a previous study (Andersson et al. 2022), which reported that despite patients having various comorbidities, diseases other than COVID‐19 were ignored, and patients were regarded solely as COVID‐19 patients, with the relationship with patients being considered unimportant. Interaction and relationship building with patients are crucial elements of person‐centred care, where nurses establish partnerships with patients, fostering a therapeutic relationship based on mutual trust (Eklund et al. 2019). This category demonstrates a collaborative relationship between nurses and patients.

Finally, in the category of *desiring stable care even in a crisis*, nurses expressed the need for systemic improvements and education to provide person‐centred care in crises such as pandemics. The participants emphasised the need for an adequate system and reinforcement of nursing staff to care for COVID‐19 patients. This aligns with the findings of a previous qualitative study on nurses' experiences during the COVID‐19 pandemic (Shahmari et al. 2023), which highlighted the issue of nursing staff shortages. During the unprecedented COVID‐19 pandemic, hospitals were not equipped with sufficient personnel to handle the situation, and the lack of competency among existing staff and the absence of a systematic framework posed significant challenges in providing care. The lack of sufficient support for nurses due to shortages in staffing and systems can lead to significant psychological issues and contribute to nurse burnout (Fernandez et al. 2020). Previous research (Tu et al. 2020) revealed that nurses caring for COVID‐19 patients experienced psychological problems—such as sleep disorders, depression and anxiety—and faced various difficulties such as insufficient knowledge about infectious diseases, shortages of facilities and equipment, lack of protocols, risk of infection transmission and increased workload (Fernandez et al. 2020; Firouzkouhi et al. 2022), which prevented them from practicing person‐centred care. When deviating from a person‐centred approach during a pandemic, patient trust can be undermined and larger issues can arise, thus resulting in a loss of the benefits gained through safe, efficient and high‐quality nursing care (Coulter and Richards 2020). Furthermore, even when providing nursing care in accordance with infection‐control guidelines during a pandemic, the consideration of person‐centred care is crucial to maintaining patients' mental health and quality of life (Dichter et al. 2020). Therefore, it is crucial to prepare optimal organisational and systematic conditions that enable nurses to practice person‐centred care even during pandemics (Andersson et al. 2022). Additionally, participants emphasised the need for training on person‐centred care. The healthcare environment, in the context of infectious diseases, faces challenges in providing person‐centred care due to its focus on disease‐centred, treatment‐oriented medical services. A paradigm shift toward person‐centred healthcare services is required in clinical practice to establish person‐centred care as a core element and change practitioners' perceptions.

### 4.1 Strengths and Limitations

While previous studies have focused on understanding the challenges faced by patients and nurses during the COVID‐19 pandemic, the present study is significant in that it focuses on how person‐centred care is practised during crises. Specifically, it explores the meaning of person‐centred care during a pandemic. Considering the possibility of similar situations arising in the future, this study's findings can be valuable in establishing policy measures to improve the implementation of person‐centred care.

This study has some limitations. First, it was conducted targeting nurses from only some hospitals in South Korea; thus, there are limitations in generalising the study's results. Second, as it did not incorporate patients' perspectives regarding person‐centred care or those of other healthcare professionals, there exists a constraint in achieving a comprehensive understanding of delivering person‐centred care in clinical settings.

### 4.2 Recommendations for Further Research

Based on the findings of this study, several suggestions can be offered. First, further research should be conducted to explore the experiences of nurses practising person‐centred care during pandemic settings, targeting nurses working in various regions and hospitals of different scales. Second, for a more profound comprehension of person‐centred care in pandemic settings, further research involving not only nurses but also patients and other healthcare professionals is required. Finally, focusing on the barriers to person‐centred care during a pandemic can lead to the exploration of more specific strategies to improve its implementation.

## 5 Conclusion

This study explored the experiences of nurses providing person‐centred care to COVID‐19 patients. Although nurses initially experienced fear, anxiety and various physical challenges—which made it difficult for them to implement person‐centred care—they gradually experienced conflict between the need to provide treatment‐centred nursing according to infection‐control guidelines and the inability to address patients' individual needs. They sought to understand and address patients' psychological and physical challenges under infection‐control measures. They also aimed to overcome COVID‐19 together with their patients, and these interactions provided satisfaction to not only the patients but also their nurses. Furthermore, nurses expressed hope for systemic improvements and education to enable the consistent practice of person‐centred care, even during a pandemic. During crises, there is a need to redesign nursing practices in a direction that respects and trusts patients through therapeutic relationships, treats patients as individuals, and partners with them to overcome diseases. Furthermore, future research examining the obstacles nurses encounter when delivering person‐centred care during a pandemic is crucial for exploring targeted strategies to strengthen this approach.

## Author Contributions

The study design: M.K., Y.L.; data collection: M.K., Y.L. and H.J.K.; data analysis and interpretation: M.K., Y.L. and H.J.K.; drafting manuscript: M.K., Y.L. and H.J.K.; revising manuscript content and editing: M.K., Y.L. and H.J.K. All authors have read and agreed to the published version of the manuscript.

## Ethics Statement

Approval for the study was obtained from the University Institutional Review Board (KWNUIRB‐2022‐03‐001‐001).

## Consent

Prior to data collection, the researchers confirmed voluntary participation through written consent.

## Conflicts of Interest

The authors declare no conflicts of interest.

## Data Availability

The data that support the findings of this study are available on request from the authors.

## References

[nop270293-bib-0001] Andersson, M. , A. Nordin , and Å. Engström . 2022. “Critical Care Nurses' Experiences of Working During the First Phase of the COVID‐19 Pandemic–Applying the Person‐Centred Practice Framework.” Intensive and Critical Care Nursing 69: 103179. 10.1016/j.iccn.2021.103179. 34895797 PMC8595352

[nop270293-bib-0002] Becqué, Y. N. , W. van der Geugten , A. Van der Heide , et al. 2022. “Dignity Reflections Based on Experiences of End‐Of‐Life Care During the First Wave of the COVID‐19 Pandemic: A Qualitative Inquiry Among Bereaved Relatives in The Netherlands (The CO‐LIVE Study).” Scandinavian Journal of Caring Sciences 36, no. 3: 769–781. 10.1111/scs.13038. 34625992 PMC8661881

[nop270293-bib-0003] Chung, S. , M. Seong , and J.‐Y. Park . 2022. “Nurses' Experience in COVID‐19 Patient Care.” Journal of the Korean Academy of Nursing Administration 28, no. 2: 142–153. 10.11111/jkana.2022.28.2.142.

[nop270293-bib-0004] Coulter, A. , and T. Richards . 2020. “Care During COVID‐19 Must Be Humane and Person Centred.” BMJ (Clinical Research Ed.) 370: m3483. 10.1136/bmj.m3483. 32900782

[nop270293-bib-0005] Dichter, M. N. , M. Sander , S. Seismann‐Petersen , and S. Köpke . 2020. “COVID‐19: It Is Time to Balance Infection Management and Person‐Centered Care to Maintain Mental Health of People Living in German Nursing Homes.” International Psychogeriatrics 32, no. 10: 1157–1160. 10.1017/S1041610220000897. 32393407 PMC7264450

[nop270293-bib-0006] Edvardsson, D. 2015. “Notes on Person‐Centred Care: What It Is and What It Is Not.” Nordic Journal of Nursing Research 35, no. 2: 65–66. 10.1177/0107408315582296.

[nop270293-bib-0007] Eklund, J. H. , I. K. Holmström , T. Kumlin , et al. 2019. ““Same Same or Different?” A Review of Reviews of Person‐Centered and Patient‐Centered Care.” Patient Education and Counseling 102, no. 1: 3–11. 10.1016/j.pec.2018.08.029. 30201221

[nop270293-bib-0008] Fernandez, R. , H. Lord , E. Halcomb , et al. 2020. “Implications for COVID‐19: A Systematic Review of Nurses' Experiences of Working in Acute Care Hospital Settings During a Respiratory Pandemic.” International Journal of Nursing Studies 111: 103637. 10.1016/j.ijnurstu.2020.103637. 32919358 PMC7206441

[nop270293-bib-0009] Firouzkouhi, M. , A. Abdollahimohammad , K. Rezaie‐Kheikhaie , et al. 2022. “Nurses' Caring Experiences in COVID‐19 Pandemic: A Systematic Review of Qualitative Research.” Health Sciences Review 3: 100030. 10.1016/j.hsr.2022.100030. 35615410 PMC9123825

[nop270293-bib-0037] Hsieh, H. F. , and E. S. Sarah . 2005. “Three Approaches to Qualitative Content Analysis.” Qualitative Health Research 15, no. 9: 1277–1288. 10.1177/1049732305276687. 16204405

[nop270293-bib-0010] Jung, T. M. , and K. A. Kim . 2022. “The Influence of Nursing Professionalism, Communication Competence and Nursing Work Environment of Dedicated COVID‐19 Hospital Nurse on Person‐Centered Care.” Journal of Korean Academic Society of Home Health Care Nursing 29, no. 2: 165–174. 10.22705/jkashcn.2022.29.2.165.

[nop270293-bib-0011] Kackin, O. , E. Ciydem , O. S. Aci , and F. Y. Kutlu . 2021. “Experiences and Psychosocial Problems of Nurses Caring for Patients Diagnosed With COVID‐19 in Turkey: A Qualitative Study.” International Journal of Social Psychiatry 67, no. 2: 158–167. 10.1177/0020764020942788. 32674644

[nop270293-bib-0012] Karimi, Z. , Z. Fereidouni , M. Behnammoghadam , et al. 2020. “The Lived Experience of Nurses Caring for Patients With COVID‐19 in Iran: A Phenomenological Study.” Risk Management and Healthcare Policy 13: 1271–1278. 10.2147/RMHP.S258785. 32904130 PMC7450521

[nop270293-bib-0013] Kim, K. 2021. “A Phenomenological Study on the Nurse's COVID‐19 Patient Care Experience in the Infectious Disease Hospitals.” Journal of Humanities and Social Science 21, no. 5: 2145–2160. 10.22143/HSS21.12.5.151.

[nop270293-bib-0014] Kim, N. , Y. Yang , and J. Ahn . 2022. “Nurses' Experiences of Care for Patients in Coronavirus Disease 2019 Infection Wards During the Early Stages of the Pandemic.” Korean Journal of Adult Nursing 34, no. 1: 109–121. 10.7475/kjan.2022.34.1.109.

[nop270293-bib-0016] Lee, J. Y. , S. Lee , and E. G. Oh . 2020. “Conceptualization of Person‐Centered Care in Korean Nursing Literature: A Scoping Review.” Korean Journal of Adult Nursing 32, no. 4: 354–363. 10.7475/kjan.2020.32.4.354.

[nop270293-bib-0015] Lee, J.‐H. , and Y. Song . 2021. “Nurses' Experiences of the COVID‐19 Crisis.” Journal of the Korean Academy of Nursing 51, no. 6: 689–702. 10.4040/jkan.21160. 35023858

[nop270293-bib-0017] Lee, S. , Y. Kim , H.‐Y. Kwon , et al. 2021. “Factors Associated With Depression During the Coronavirus Disease 2019 (COVID‐19) Quarantine in Four Districts of Seoul Metropolitan City.” Korean Journal of Rehabilitation Nursing 24, no. 1: 15–24. 10.7587/kjrehn.2021.15.

[nop270293-bib-0018] Li, J. , J. Wang , X. Kong , et al. 2021. “Person‐Centered Communication Between Health Care Professionals and COVID‐19‐Infected Older Adults in Acute Care Settings: Findings From Wuhan, China.” Journals of Gerontology: Series B 76, no. 4: e225–e229. 10.1093/geronb/gbaa190. PMC7665773 33136158

[nop270293-bib-0019] Lusk, J. M. , and K. Fater . 2013. “A Concept Analysis of Patient‐Centered Care.” Nursing Forum 48, no. 2: 89–98. 10.1111/nuf.12019. 23600637

[nop270293-bib-0020] Manookian, A. , N. D. Nayeri , S. Dashti , and M. Shahmari . 2024. “Ethical Challenges to the Self‐Care of Nurses During the Covid‐19 Pandemic.” Nursing Ethics 31, no. 2–3: 161–175. 10.1177/09697330231180753. 37428123

[nop270293-bib-0021] Martin, A. 2022. “Overcoming COVID‐19 Constraints on Person Centered Dementia Care: A Narrative Inquiry of Lived Experiences of Residential Care Staff in Belgium.” Journal of Long‐Term Care: 183–192. 10.31389/jltc.129.

[nop270293-bib-0022] Morgan, J. C. , W. Ahmad , Y.‐Z. Chen , and E. O. Burgess . 2023. “The Impact of COVID‐19 on the Person‐Centered Care Practices in Nursing Homes.” Journal of Applied Gerontology 42, no. 7: 1582–1587. 10.1177/07334648231154544. 36727467 PMC9899667

[nop270293-bib-0023] Noh, E.‐Y. , J. Y. Chai , H. J. Kim , E. Kim , and P. Yeon‐Hwan . 2021. “Nurses' Experience With Caring for COVID‐19 Patients in a Negative Pressure Room Amid the Pandemic Situation.” Journal of the Korean Academy of Nursing 51, no. 5: 585–596. 10.4040/jkan.21148. 34737251

[nop270293-bib-0024] Oh, H. , and N. K. Lee . 2021. “A Phenomenological Study of the Lived Experience of Nurses Caring for Patients With COVID‐19 in Korea.” Journal of Korean Academy of Nursing 51, no. 5: 561–572. 10.4040/jkan.21112. 34737249

[nop270293-bib-0025] Oh, I. O. , S. J. Yoon , and K. A. Nam . 2021. “Working Experience of Nurses at a COVID‐19 Dedicated Hospital.” Korean Journal of Adult Nursing 33, no. 6: 657–669. 10.7475/kjan.2021.33.6.657.

[nop270293-bib-0026] Rao, A. , and A. Kelemen . 2021. “Lessons Learned From Caring for Patients With COVID‐19 at the End of Life.” Journal of Palliative Medicine 24, no. 3: 468–471. 10.1089/jpm.2020.0251. 32833568

[nop270293-bib-0027] Robinson, R. , and C. K. Stinson . 2021. “The Lived Experiences of Nurses Working During the COVID‐19 Pandemic.” Dimensions of Critical Care Nursing 40, no. 3: 156–163. 10.1097/DCC.0000000000000481. 33792274 PMC8030877

[nop270293-bib-0028] Safdari, A. , M. Rassouli , M. Elahikhah , et al. 2023. “Explanation of Factors Forming Missed Nursing Care During the COVID‐19 Pandemic: A Qualitative Study.” Frontiers in Public Health 11: 989458. 10.3389/fpubh.2023.989458. 36778543 PMC9909100

[nop270293-bib-0029] Shahmari, M. , N. D. Nayeri , A. Palese , S. Dashti , and A. Manookian . 2024. “Seeking Protection in the Heart of the Storm: Findings From a Grounded Theory Study.” Journal of Nursing Management 2024, no. 1: 6185455. 10.1155/2024/6185455. 40224868 PMC11918800

[nop270293-bib-0030] Shahmari, M. , N. D. Nayeri , A. Palese , and A. Manookian . 2023. “Nurses' Safety‐Related Organisational Challenges During the COVID‐19 Pandemic: A Qualitative Study.” International Nursing Review 70, no. 1: 18–27. 10.1111/inr.12811. 36515574 PMC9877888

[nop270293-bib-0031] Shin, J. W. , and M. K. Cheon . 2021. “Expansion of Healthcare Resources for COVID‐19 Response: Three Axes.” Health and Welfare Issue & Focus 408: 1–11.

[nop270293-bib-0032] Tong, A. , P. Sainsbury , and J. Craig . 2007. “Consolidated Criteria for Reporting Qualitative Research (COREQ): A 32‐Item Checklist for Interviews and Focus Groups.” International Journal for Quality in Health Care 19, no. 6: 349–357. 10.1093/intqhc/mzm042. 17872937

[nop270293-bib-0033] Tu, Z.‐h. , J.‐w. He , and N. Zhou . 2020. “Sleep Quality and Mood Symptoms in Conscripted Frontline Nurse in Wuhan, China During COVID‐19 Outbreak: A Cross‐Sectional Study.” Medicine 99, no. 26: e20769. 10.1097/MD.0000000000020769. 32590755 PMC7328950

[nop270293-bib-0034] Williams, L. A. , D. Accardo , J. Dolgoff , et al. 2024. “A Mixed Methods Study: The Grief Experience of Registered Nurses Working on the Frontlines During the COVID‐19 Pandemic.” Journal of Clinical Nursing 33, no. 1: 344–356. 10.1111/jocn.16579. 36352533 PMC9878071

[nop270293-bib-0035] World Health Organization . 2022. World Health Organization (WHO) Coronavirus (COVID‐19) Dashboard (2022) Accessed June 21, 2023. https://covid19.who.int.

[nop270293-bib-0036] Yun, H.‐J. , and J. Jeon . 2022. “Factors Influencing the Performance of Person‐Centered Care Among Nurses in Designated COVID‐19 Hospitals.” Korean Journal of Adult Nursing 34, no. 4: 413–423. 10.7475/kjan.2022.34.4.413.

